# Outcomes associated with anaesthetic techniques for caesarean section in low- and middle-income countries: a secondary analysis of WHO surveys

**DOI:** 10.1038/s41598-020-66897-8

**Published:** 2020-06-23

**Authors:** Pisake Lumbiganon, Hla Moe, Siriporn Kamsa-ard, Siwanon Rattanakanokchai, Malinee Laopaiboon, Chumnan Kietpeerakool, Nampet Jampathong, Monsicha Somjit, José Guilherme Cecatti, Joshua P. Vogel, Ana Pilar Betran, Suneeta Mittal, Maria Regina Torloni

**Affiliations:** 1 Department of Obstetrics and Gynaecology, Faculty of Medicine, Khon Kaen University, 123 Mitraparb Road, Amphur Muang Khon Kaen, 40002 Thailand; 2 Department of Preventive and Social Medicine, University of Medicine, 30th Street, Between 73rd and 74th Streets, Mandalay, Myanmar; 3 Department of Epidemiology and Biostatistics, Faculty of Public Health, Khon Kaen University, 123 Mitraparb Road, Amphur Muang Khon Kaen, 40002 Thailand; 4 Cochrane Thailand, Faculty of Medicine, Khon Kaen University, 123 Mitraparb Road, Amphur Muang Khon Kaen, 40002 Thailand; 5 Department of Anesthesia, Faculty of Medicine, Khon Kaen University, 123 Mitraparb Road, Amphur Muang Khon Kaen, 40002 Thailand; 6 Department of Ginecology and Obstetrics, School of Medical Sciences, Universidade de Campinas, Campinas, SP Brazil; 7 UNDP/UNFPA/UNICEF/WHO/World Bank Special Programme of Research, Development and Research Training in Human Reproduction (HRP), Department of Sexual and Reproductive Health and Research, World Health Organization, Avenue Appia 20, Geneva, CH-1211 Switzerland; 8 Department of Obstetrics and Gynaecology, Fortis Memorial Research Institute, Gurugram, 122002 India; 9 Evidence Based Healthcare Post Graduate Programme, Department of Medicine, São Paulo Federal University, Rua Botucatu 740, 3o andar, São Paulo, SP, CEP 04023-900 Brazil

**Keywords:** Health occupations, Medical research

## Abstract

Associations between anaesthetic techniques and pregnancy outcomes were assessed among 129,742 pregnancies delivered by caesarean section (CS) in low- and middle-income countries (LMICs) using two WHO databases. Anaesthesia was categorized as general anaesthesia (GA) and neuraxial anaesthesia (NA). Outcomes included maternal death (MD), maternal near miss (MNM), severe maternal outcome (SMO), intensive care unit (ICU) admission, early neonatal death (END), neonatal near miss (NNM), severe neonatal outcome (SNO), Apgar score <7 at 5 minutes, and neonatal ICU (NICU) admission. A two‐stage approach of individual participant data meta‐analysis was used to combine the results. Adjusted odds ratio (OR) with 95% confidence intervals (CIs) were presented. Compared to GA, NA were associated with decreased odds of MD (pooled OR 0.28; 95% CI 0.10, 0.78), MNM (pooled OR 0.25; 95% CI 0.21, 0.31), SMO (pooled OR 0.24; 95% CI 0.20,0.28), ICU admission (pooled OR 0.17; 95% CI 0.13, 0.22), NNM (pooled OR 0.63; 95% CI 0.55, 0.73), SNO (pooled OR 0.55; 95% CI 0.48, 0.63), Apgar score <7 at 5 minutes (pooled OR 0.35; 95% CI 0.29, 0.43), and NICU admission (pooled OR 0.53; 95% CI 0.45, 0.62). NA therefore was associated with decreased odds of adverse pregnancy outcomes in LMICs.

## Introduction

Caesarean section (CS) can be a life-saving procedure for women and babies when potentially life-threatening complications occur during pregnancy or childbirth, such as abnormal fetal presentation, non-reassuring foetal condition, abnormal placentation, obstetric haemorrhage, and obstructed labor^1^.

CS can be performed under either neuraxial anaesthesia (NA) including spinal anaesthesia (SA) and epidural anaesthesia (EA), or general anaesthesia (GA). The choice of anaesthesia for CS generally depends on clinical indications, experience of the anaesthesiologist, as well as maternal preferences. NA offers the benefit of the woman being awake during the procedure, with minimal anaesthetic exposure to the neonate. NA also lessens the risks of maternal aspiration and difficult airway associated with GA. In general, NA can be used for more than 90% of women undergoing CS^2^. Certain conditions contraindicate the use of NA, including infection at the needle insertion site, significant coagulopathy, hypovolaemic shock, increased intracranial pressure from a space-occupying lesion and inadequate provider expertise^2^. GA is generally used for CS when NA is contraindicated or for emergent CS because of its rapid and predictable effect^2^. Previous systematic reviews and meta-analyses of randomised controlled trials (RCTs) reported that NA was associated with lower estimated maternal blood loss compared to GA. GA was, however, superior to NA in terms of women satisfaction^3,4^.

The rate of maternal deaths (MD) following CS is notably high in many low- and middle-income countries (LMICs). The estimated rate of MD in women who had a CS in LMIC has been estimated at 7.6 per 1000 procedures, with one-fourth of MD occurring in women who had undergone a CS^5^. A previous systematic review conducted to assess anaesthesia-attributed deaths of pregnant women in LMICs reported that anaesthesia accounted for 2.8% of all MD and 13.8% of MD after CS^6^. Furthermore, exposure to GA was associated with increased odds of maternal and perinatal deaths, compared with NA^6^. This systematic review, however, had important limitations due to differences in methodological quality, outcome measures, and outcome definitions applied across the included studies. In addition, most of the included studies were from sub-Saharan Africa and thus may not represent an overview of LMICs^6^. We therefore performed this secondary analysis to assess the association between anaesthetic technique for CS and adverse pregnancy outcomes in LMICs using the two large WHO databases - The World Health Organization Global Survey (WHOGS) on Maternal and Perinatal Health^7^ and The World Health Organization Multi-Country Survey (WHOMCS) on Maternal and Newborn Health^8^.

## Results

### Characteristics of study population

**Figure 1:**
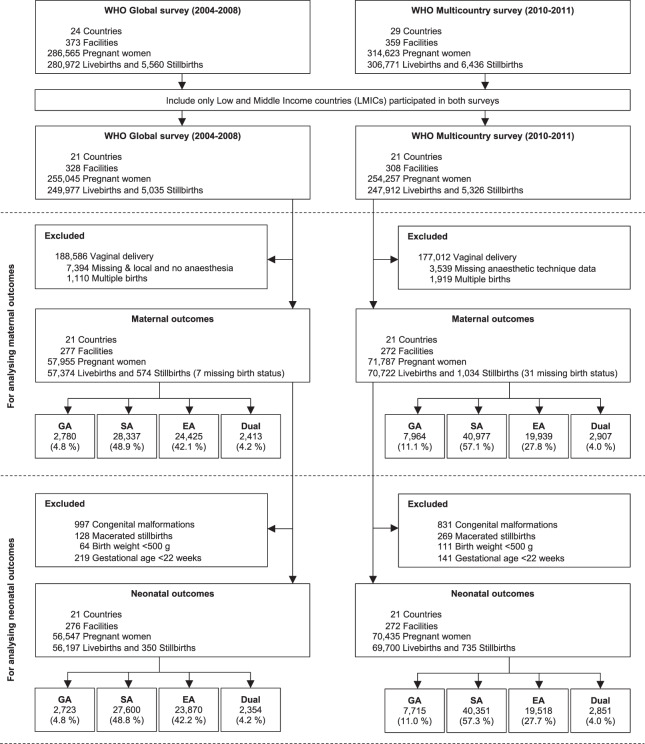
Study flow diagram.

We included 129,742 women from WHOGS and WHOMCS for the analyses of maternal outcomes (Fig. 1). The analyses of neonatal outcomes consisted of 125,897 livebirths and 1085 stillbirths. Spinal anaesthesia was the most common anaesthetic technique in both databases accounting for 48.9% in WHOGS and 57.1% in WHOMCS. The rate of GA was roughly twice as high for women in WHOMCS as for those in WHOGS. Approximately 4% of women in either WHOGS or WHOMCS were recorded to receive more than one anaesthetic technique.

Table 1 displays the baseline characteristics of women and newborns included in this study. Maternal and neonatal characteristics were similar in the two surveys in terms of maternal age, education, marital status, parity, gestational age, infant sex and birthweight. Comorbidity noted among women included in WHOGS and WHOMCS was 30.6% and 11.7%, respectively. Data regarding comorbidity however was not available in approximately 32% of women included in WHOGS compared to 0.1% of data obtained from WHOMCS.

**Table 1:** Characteristics of mothers and newborns by sources of data. Note: WHOGS = World Health Organization Global Survey on maternal and perinatal health, WHOMCS = World Health Organization Multi-Country Survey on maternal and newborn health, NA = not available.

Baseline characteristics	WHOGS		WHOMCS	
Maternal characteristics	n (%)		n (%)	
Age (years)	(n = 57,955)		(n = 71,787)	
<20	5,277	(9.1)	6,327	(8.8)
20–34	44,964	(77.6)	54,824	(76.4)
≥ 35	7,681	(13.2)	10,513	(14.6)
Missing	33	(0.1)	123	(0.2)
**Years of education**				
0–6	12,750	(22.0)	11,709	(16.3)
7–12	32,187	(55.6)	37,280	(51.9)
> 12	10,515	(18.1)	17,394	(24.3)
Missing	2,503	(4.3)	5,404	(7.5)
**Marital status**				
No partner	7,163	(12.4)	7,786	(10.9)
With partner	50,675	(87.4)	63,489	(88.4)
Missing	117	(0.2)	512	(0.7)
**No of previous births**				
0	27,170	(47.0)	35,930	(50.0)
1	18,453	(31.8)	21,153	(29.5)
>1	12,195	(21.0)	14,582	(20.3)
Missing	137	(0.2)	122	(0.2)
**Gestational age (weeks)**				
<37	5,804	(10.0)	6351	(8.9)
≥37	51,917	(89.6)	65282	(90.9)
Missing	234	(0.4)	154	(0.2)
**Comorbidity**				
Present	17,734	(30.6)	8,415	(11.7)
Absent	21,807	(37.6)	63,368	(88.3)
Missing	18,414	(31.8)	4	(0.01)
**Type of CS**				
Ante-partum	35,166	(60.7)	28,484	(39.7)
Intra-partum	22,789	(39.3)	43,256	(60.3)
Missing	0	(0.0)	47	(0.1)
**Anaesthesia providers**				
Anaesthetist	41,762	(72.1)		NA
Others	15,647	(27.0)		NA
Missing	546	(0.9)		NA
Neonatal characteristics	(**n = 56,547)**		**(n = 70,435)**	
**Sex**				
Female	26,696	(47.2)	33,222	(47.2)
Male	29,825	(52.7)	37,138	(52.7)
Missing	26	(0.1)	75	(0.1)
**Birth weight (grams)**				
<2500	5,968	(10.6)	8,128	(11.5)
≥2500	50,579	(89.4)	62,307	(88.5)
Missing	0	(0.0)	0	(0.0)

The rate of intrapartum CS was much higher in WHOMCS than that in WHOGS (60.3% versus 39.3%, respectively). In WHOGS, approximately 70% of anaesthesia was provided by anaesthetists. Data regarding the types of anaesthesia providers was not available in WHOMCS (Table 1).

### Adverse maternal and neonatal outcomes

Table 2 shows the rates of adverse maternal and neonatal outcomes by sources of data and type of anaesthesia. The maternal death (MD) rate was 0.1% for both databases. Maternal near miss (MNM) was 7.7% and 1.0% for women in WHOGS and WHOMCS, respectively. Early neonatal death (END) varied from 0.7% in WHOGS to 0.9% in WHOMCS. Neonatal near miss (NNM) rate in WHOGS and WHOMCS was 3.8% and 9.4%, respectively.

**Table 2:** Adverse maternal and neonatal outcomes by anaesthetic technique for CS in WHOGS and WHOMCS. Note: GA = general anaesthesia, SA = spinal anaesthesia, EA = epidural anaesthesia, ICU = intensive care unit, NICU = neonatal intensive care unit.

WHO Global survey								
Maternal outcomes	GA (n = 2,780)		SA (n = 28,337)		EA (n = 24,425)		>1 technique (n = 2,413)	
	n	(%)	n	(%)	n	(%)	n	(%)
Maternal death	9	(0.3)	38	(0.1)	6	(0.1)	6	(0.2)
Missing	0	(0.0)	22	(0.1)	6	(0.1)	1	(0.1)
Maternal near miss	298	(10.7)	2944	(10.4)	1000	(4.1)	227	(9.4)
Missing	0	(0.0)	65	(0.2)	275	(1.1)	3	(0.1)
Severe maternal outcomes	307	(11.0)	2982	(10.5)	1006	(4.1)	233	(9.7)
Missing	0	(0.0)	65	(0.2)	275	(1.1)	3	(0.1)
Admission to ICU	160	(5.8)	1915	(6.8)	389	(1.6)	154	(6.4)
Missing	0	(0.0)	10	(0.1)	23	(0.1)	0	(0.0)
Postpartum haemorrhage	55	(2.0)	199	(0.7)	95	(0.4)	24	(1.0)
Missing	0	(0.0)	15	(0.1)	10	(0.1)	0	(0.0)
**Neonatal outcomes**	**GA** **(n** = **2,723)**		**SA** **(n** = **27,600)**		**EA** **(n** = **23,870)**		**>1 technique** **(n** = **2,354)**	
	**n**	**(%)**	**n**	**(%)**	**n**	**(%)**	**n**	**(%)**
Early neonatal death	18	(0.7)	238	(0.9)	138	(0.6)	16	(0.7)
Missing	36	(1.3)	231	(0.8)	81	(0.3)	28	(1.2)
Neonatal near miss	127	(4.7)	1012	(3.7)	911	(3.8)	78	(3.3)
Missing	36	(1.3)	271	(1.0)	90	(0.4)	34	(1.4)
Severe neonatal outcome	145	(5.3)	1250	(4.5)	1049	(4.4)	94	(4.0)
Missing	36	(1.3)	271	(1.0)	90	(0.4)	34	(1.4)
Apgar score <7 at 5 mins	86	(3.2)	670	(2.4)	437	(1.8)	55	(2.3)
Missing	34	(1.2)	256	(0.9)	87	(0.4)	34	(1.4)
Admission to ICU	365	(13.4)	4684	(17.0)	3180	(13.3)	243	(10.3)
Missing	34	(1.2)	218	(0.8)	85	(0.4)	28	(1.2)
WHO Multicountry survey								
**Maternal outcomes**	**GA** **(n** = **7,964)**		**SA** **(n** = **40,977)**		**EA** **(n** = **19,939)**		**>1 technique** **(n** = **2,907)**	
	**n**	**(%)**	**n**	**(%)**	**n**	**(%)**	**n**	**(%)**
Maternal death	53	(0.7)	38	(0.1)	5	(0.1)	4	(0.1)
Missing	0	(0.0)	0	(0.0)	0	(0.0)	0	(0.0)
Maternal near miss	282	(3.5)	248	(0.6)	147	(0.7)	32	(1.1)
Missing	4	(0.1)	33	(0.1)	118	(0.6)	2	(0.1)
Severe maternal outcomes	335	(4.2)	286	(0.7)	152	(0.8)	36	(1.2)
Missing	4	(0.1)	33	(0.1)	118	(0.6)	2	(0.1)
Admission to ICU	185	(2.3)	257	(0.6)	268	(1.3)	41	(1.5)
Missing	2	(0.1)	16	(0.1)	31	(0.2)	1	(0.1)
Postpartum haemorrhage	308	(3.9)	516	(1.3)	259	(1.3)	97	(3.3)
Missing	1	(0.1)	5	(0.1)	3	(0.1)	0	(0.1)
**Neonatal outcomes**	**GA** **(n** = **7,715)**		**SA** **(n** = **40,351)**		**EA** **(n** = **19,518)**		**>1 technique** **(n** = **2,851)**	
	**n**	**(%)**	**n**	**(%)**	**n**	**(%)**	**n**	**(%)**
Early neonatal death	145	(1.9)	379	(0.9)	91	(0.5)	8	(0.3)
Missing	298	(3.9)	390	(1.0)	76	(0.4)	20	(0.7)
Neonatal near miss	963	(12.5)	3779	(9.4)	1703	(8.7)	150	(5.3)
Missing	322	(4.2)	557	(1.4)	179	(0.9)	26	(0.9)
Severe neonatal outcome	1108	(14.4)	4158	(10.3)	1794	(9.2)	158	(5.5)
Missing	322	(4.2)	557	(1.4)	179	(0.9)	26	(0.9)
Apgar score <7 at 5 min	457	(5.9)	1048	(2.6)	188	(1.0)	44	(1.5)
Missing	303	(3.9)	515	(1.3)	75	(0.4)	19	(0.7)
Admission to NICU	897	(11.6)	4265	(10.6)	2081	(10.7)	162	(5.7)
Missing	296	(3.8)	371	(0.9)	59	(0.3)	21	(0.7)

### Association between anaesthetic technique for CS and maternal outcomes

**Figure 2:**
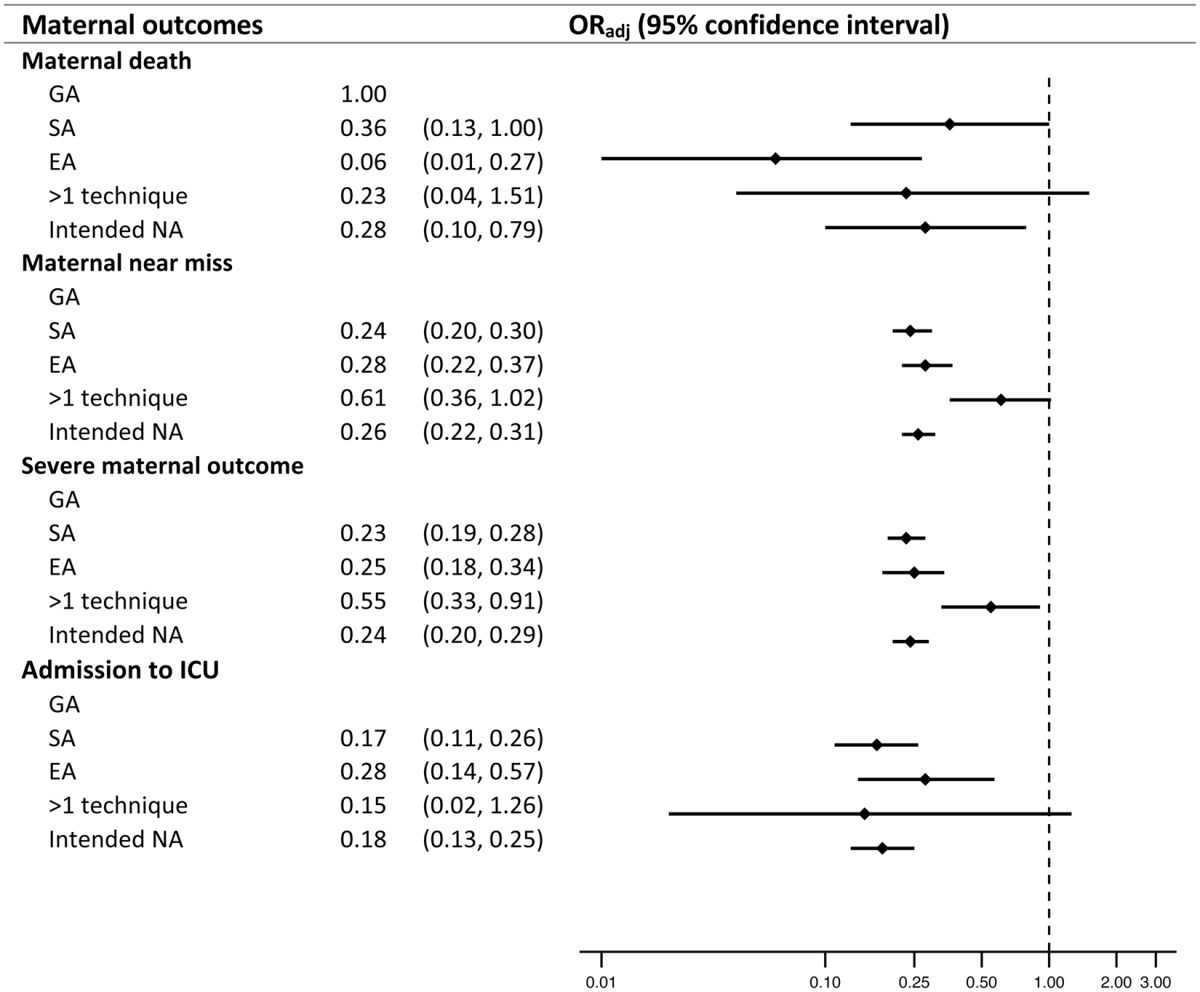
Forest plot of the pooled estimates of maternal outcomes from WHOGS and WHOMCS datasets. Note: GA = general anaesthesia, SA = spinal anaesthesia, EA = epidural anaesthesia, NA = neuraxial anaesthesia, NICU = neonatal intensive care unit. Intended NA included data of women undergoing SA and EA and those who received >1 type of anaesthesia Intended NA included data of women undergoing SA and EA and those who received >1 type of anaesthesia.

The analyses for both surveys show that NA was associated with significantly lower odds of MD, MNM, severe maternal outcome (SMO), admission to intensive care unit (ICU) and postpartum haemorrhage (PPH). These associations were consistent for both antepartum and intrapartum CS. For WHOGS, the benefit in reducing the odds of MD was only seen in antepartum CS (Table 3). In WHOMCS, the decreased odds of MD in women undergoing NA was observed regardless of the timing of CS performed (Table 4). Figure 2 demonstrates the pooled estimates of associated risk of anaesthesia for individual maternal outcome. NA was associated with reduced odds of MD (pooled OR 0.28; 95% CI 0.10, 0.78), MNM (pooled OR 0.25; 95% CI 0.21, 0.31), SMO (pooled OR 0.24; 95% CI 0.20, 0.28), and ICU admission (pooled OR 0.17; 95% CI 0.13, 0.22). Women receiving more than one anaesthetic technique during CS carried similar odds of MD (pooled OR 0.23; 95% CI 0.04, 1.51), MNM (pooled OR 0.61; 95% CI 0.36, 1.02), and ICU admission (pooled OR 0.15; 95% CI 0.02, 1.26) to those with GA (Fig. 2).

**Table 3:** Associations between anaesthetic techniques for caesarean section and pregnancy outcomes according the time of performance of CS in WHOGS. Note:.

Outcomes	WHO Global survey					
	All		Antepartum CS		Intrapartum CS	
	OR_Adj_	(95% CI)	OR_Adj_	(95% CI)	OR_Adj_	(95% CI)
**Maternal outcomes**						
Maternal death						
GA	1.00		1.00		1.00	
SA	0.70	(0.21, 2.31)^a^	0.12	(0.02, 0.90)^c^	1.51	(0.27, 8.41)^c^
EA	0.14	(0.02, 0.87)^a^	0.06	(0.00, 0.89)^c^	0.19	(0.01, 2.70)^c^
>1 technique	Not estimable		Not estimable		Not estimable	
Maternal near miss						
GA	1.00		1.00		1.00	
SA	0.23	(0.18, 0.30)^a^	0.26	(0.18, 0.36)^c^	0.20	(0.13, 0.33)^c^
EA	0.31	(0.22, 0.44)^a^	0.33	(0.22, 0.50)^c^	0.20	(0.11, 0.37)^c^
>1 technique	0.55	(0.28, 1.08)^a^	0.61	(0.29, 1.28)^c^	0.21	(0.05, 0.86)^c^
Severe maternal outcome						
GA	1.00		1.00		1.00	
SA	0.22	(0.17, 0.29)^a^	0.25	(0.18, 0.35)^c^	0.19	(0.12, 0.31)^c^
EA	0.29	(0.21, 0.42)^a^	0.32	(0.21, 0.48)^c^	0.20	(0.11, 0.37)^c^
>1 technique	0.51	(0.26, 0.99)^a^	0.57	(0.27, 1.19)^c^	0.20	(0.05, 0.84)^c^
Admission to ICU						
GA	1.00		1.00		1.00	
SA	0.22	(0.13, 0.36)^a^	0.28	(0.15, 0.52)^c^	0.13	(0.05, 0.31)^c^
EA	0.42	(0.22, 0.80)^a^	0.43	(0.19, 0.96)^c^	0.33	(0.11, 0.98)^c^
>1 technique	0.08	(0.02, 0.35)^a^	0.11	(0.03, 0.50)^c^	0.10	(0.00, 3.95)^c^
Postpartum haemorrhage requiring blood transfusion						
GA	1.00		1.00		1.00	
SA	0.35	(0.21, 0.58)^a^	0.32	(0.17, 0.60)^c^	0.39	(0.17, 0.88)^c^
EA	0.29	(0.15, 0.58)^a^	0.24	(0.11, 0.54)^c^	0.32	(0.11, 0.97)^c^
>1 technique	0.65	(0.23, 1.87)^a^	0.60	(0.18, 1.99)^c^	0.81	(0.14, 4.52)^c^
**Neonatal outcomes**						
Early neonatal death						
GA	1.00		1.00		1.00	
SA	0.94	(0.49, 1.79)^b^	1.13	(0.49, 2.63)^d^	1.04	(0.39, 2.78)^d^
EA	0.62	(0.29, 1.31)^b^	0.87	(0.34, 2.20)^d^	0.49	(0.14, 1.68)^d^
>1 technique	0.95	(0.30, 3.08)^b^	0.96	(0.23, 3.99)^d^	0.98	(0.14, 6.78)^d^
Neonatal near miss						
GA	1.00		1.00		1.00	
SA	0.62	(0.44, 0.88)^b^	0.71	(0.47, 1.08)^\^	0.59	(0.34, 1.01)^d^
EA	0.54	(0.36, 0.80)^b^	0.60	(0.38, 0.95)^d^	0.47	(0.25, 0.90)^d^
>1 technique	0.38	(0.20, 0.74)^b^	0.35	(0.16, 0.75)^d^	0.43	(0.15, 1.24)^d^
**Severe neonatal outcome**						
GA	1.00		1.00		1.00	
SA	0.59	(0.42, 0.82)^b^	0.68	(0.45, 1.03)^d^	0.57	(0.34, 0.96)^d^
EA	0.49	(0.33, 0.72)^b^	0.57	(0.36, 0.90)^d^	0.42	(0.22, 0.80)^d^
>1 technique	0.45	(0.23, 0.86)^b^	0.39	(0.18, 0.85)^d^	0.48	(0.17, 1.35)^d^
Apgar score < 7 at 5 min						
GA	1.00		1.00		1.00	
SA	0.37	(0.25, 0.55)^b^	0.46	(0.27, 0.28)^d^	0.41	(0.23, 0.74)^d^
EA	0.30	(0.18, 0.51)^b^	0.38	(0.20, 0.72)^d^	0.24	(0.11, 0.52)^d^
>1 technique	0.39	(0.17, 0.90)^b^	0.27	(0.09, 0.85)^d^	0.47	(0.15, 1.43)^d^
Admission to NICU						
GA	1.00		1.00		1.00	
SA	0.58	(0.44, 0.75)^b^	0.61	(0.44, 0.85)^d^	0.61	(0.41, 0.93)^d^
EA	0.68	(0.49, 0.94)^b^	0.67	(0.45, 0.99)^d^	0.71	(0.42, 1.19)^d^
>1 technique	0.44	(0.23, 0.81)^b^	0.57	(0.27, 1.18)^d^	0.29	(0.10, 0.79)^d^

^a^Models for maternal outcomes of all women: adjusted for maternal age, education level, marital status, parity, gestational age, comorbid conditions, type of caesarean section (antepartum or intrapartum), type of anaesthesia provider, and facility capacity index.

^b^Models for neonatal outcomes of all neonates: adjusted for the same factors as in model 1 plus infant gender and birth weight.

^c^Models for maternal outcomes of women with antepartum or intrapartum CS: adjusted for the same factors as in model 1 but without type of caesarean section (antepartum or intrapartum).

^d^Models for neonatal outcomes of neonates born by antepartum or intrapartum CS: adjusted for the same factors as in model 2 but without type of caesarean section (antepartum or intrapartum).

CS, caesarean section; GA, general anaesthesia; SA, spinal anaesthesia; EA, epidural anaesthesia; ICU, intensive care unit; NICU, neonatal intensive care uni.t

**Table 4:** Associations between anaesthetic techniques for caesarean section and pregnancy outcomes according the time of performance of C-section in WHOMCS. Note:.

Outcomes	WHO Multicountry survey					
	All		Antepartum CS		Intrapartum CS	
	OR_Adj_	(95% CI)	OR_Adj_	(95% CI)	OR_Adj_	(95% CI)
**Maternal outcomes**						
Maternal death						
GA	1.00		1.00		1.00	
SA	0.24	(0.13, 0.45)^a^	0.20	(0.08, 0.50)^c^	0.26	(0.11, 0.60)^c^
EA	0.03	(0.01, 0.14)^a^	0.03	(0.00, 0.31)^c^	0.03	(0.00, 0.32)^c^
>1 technique	0.39	(0.08, 1.99)^a^	0.27	(0.03, 2.65)^c^	0.61	(0.06, 5.70)^c^
Maternal near miss						
GA	1.00		1.00		1.00	
SA	0.26	(0.20, 0.34)^a^	0.26	(0.18, 0.38)^c^	0.27	(0.19, 0.38)^c^
EA	0.25	(0.17, 0.37)^a^	0.26	(0.16, 0.44)^c^	0.24	(0.14, 0.42)^c^
>1 technique	0.76	(0.43, 1.33)^a^	0.65	(0.32, 1.31)^c^	0.89	(0.38, 2.10)^c^
**Severe maternal outcome**						
GA	1.00		1.00		1.00	
SA	0.24	(0.19, 0.31)^a^	0.23	(0.16, 0.33)^c^	0.26	(0.19, 0.37)^c^
EA	0.21	(0.15, 0.32)^a^	0.21	(0.13, 0.35)^c^	0.23	(0.13, 0.38)^c^
>1 technique	0.72	(0.42, 1.24)^a^	0.58	(0.29, 1.14)^c^	0.89	(0.39, 2.04)^c^
Admission to ICU						
GA	1.00		1.00		1.00	
SA	0.14	(0.10, 0.20)^a^	0.11	(0.07, 0.19)^c^	0.24	(0.15, 0.41)^c^
EA	0.20	(0.13, 0.31)^a^	0.18	(0.10, 0.32)^c^	0.38	(0.20, 0.70)^c^
>1 technique	0.20	(0.10, 0.39)^a^	0.14	(0.06, 0.31)^c^	0.53	(0.18, 1.55)^c^
Postpartum haemorrhage						
GA	1.00		1.00		1.00	
SA	0.36	(0.28, 0.45)^a^	0.39	(0.28, 0.55)^c^	0.39	(0.29, 0.53)^c^
EA	0.46	(0.34, 0.62)^a^	0.50	(0.33, 0.76)^c^	0.48	(0.32, 0.72)^c^
>1 technique	0.80	(0.53, 1.22)^a^	0.87	(0.51, 1.49)^c^	0.99	(0.53, 1.87)^c^
**Neonatal outcomes**						
Early neonatal death						
GA	1.00		1.00		1.00	
SA	0.52	(0.38, 0.71)^b^	0.60	(0.37, 0.97)^d^	0.50	(0.34, 0.75)^d^
EA	0.32	(0.20, 0.53)^b^	0.39	(0.20, 0.79)^d^	0.27	(0.14, 0.49)^d^
>1 technique	0.26	(0.09, 0.74)^b^	0.14	(0.03, 0.74)^d^	0.34	(0.09, 1.23)^d^
Neonatal near miss						
GA	1.00		1.00		1.00	
SA	0.66	(0.56, 0.77)^b^	0.71	(0.56, 0.90)^d^	0.66	(0.53, 0.82)^d^
EA	0.60	(0.48, 0.75)^b^	0.62	(0.46, 0.82)^d^	0.65	(0.49, 0.86)^d^
>1 technique	0.53	(0.38, 0.73)^b^	0.55	(0.36, 0.84)^d^	0.43	(0.26, 0.71)^d^
**Severe neonatal outcome**						
GA	1.00		1.00		1.00	
SA	0.55	(0.47, 0.65)^b^	0.60	(0.47, 0.76)^d^	0.58	(0.48, 0.72)^d^
EA	0.52	(0.42, 0.65)^b^	0.52	(0.39, 0.69)^d^	0.58	(0.44, 0.77)^d^
>1 technique	0.46	(0.33, 0.65)^b^	0.45	(0.29, 0.68)^d^	0.42	(0.25, 0.68)^d^
Apgar score < 7 at 5 min						
GA	1.00		1.00		1.00	
SA	0.37	(0.29, 0.46)^b^	0.46	(0.32, 0.68)^d^	0.35	(0.26, 0.47)^d^
EA	0.27	(0.18, 0.40)^b^	0.32	(0.18, 0.57)^d^	0.23	(0.14, 0.37)^d^
>1 technique	0.50	(0.28, 0.90)^b^	0.23	(0.08, 0.65)^d^	0.62	(0.30, 1.26)^d^
Admission to NICU						
GA	1.00		1.00		1.00	
SA	0.52	(0.44, 0.62)^b^	0.52	(0.40, 0.68)^d^	0.57	(0.46, 0.71)^d^
EA	0.45	(0.36, 0.56)^b^	0.38	(0.28, 0.53)^d^	0.56	(0.42, 0.75)^d^
>1 technique	0.40	(0.28, 0.57)^b^	0.35	(0.22, 0.56)^d^	0.39	(0.23, 0.67)^d^

^a^Models for maternal outcomes of all women: adjusted for maternal age, education level, marital status, parity, gestational age, comorbid conditions, type of caesarean section (antepartum or intrapartum), and facility capacity index.

^b^Models for neonatal outcomes of all neonates: adjusted for the same factors as in model 1 plus infant gender, birth weight.

^c^Models for maternal outcomes of women with antepartum or intrapartum CS: adjusted for the same factors as in model 1 but without type of caesarean section (antepartum or intrapartum).

^d^Models for neonatal outcomes of neonates born by antepartum or intrapartum CS: adjusted for the same factors as in model 2 but without type of caesarean section (antepartum or intrapartum).

CS = caesarean section, GA = general anaesthesia, SA = spina anaesthesia, EA = epidural anaesthesia, ICU = intensive care unit, NICU = neonatal intensive care unit.

### Association between anaesthetic technique for CS and neonatal outcomes

**Figure 3:**
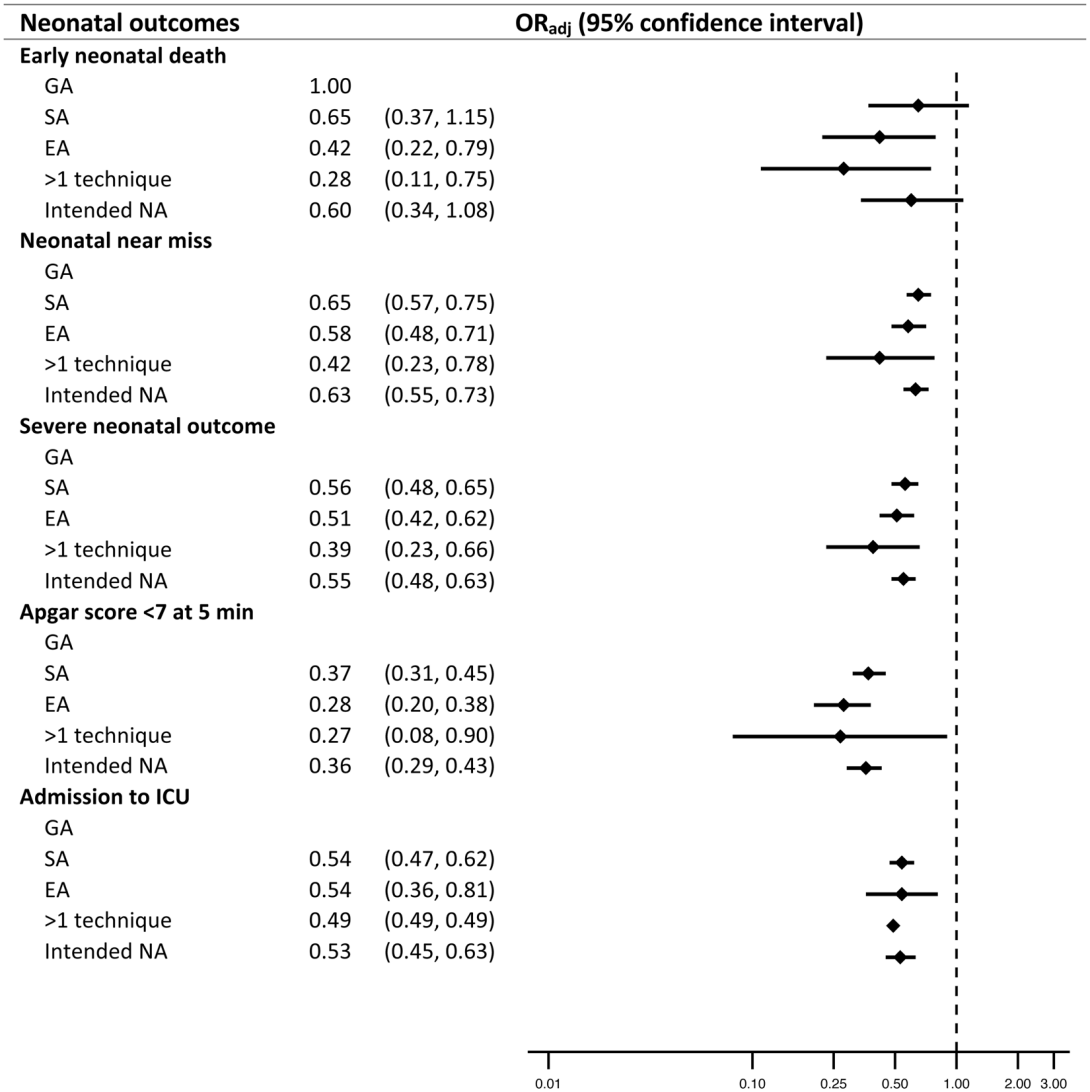
Forest plot of the pooled estimates of neonatal outcomes from WHOGS and WHOMCS datasets. Note: GA = general anaesthesia, SA = spinal anaesthesia, EA = epidural anaesthesia, NA = neuraxial anaesthesia, NICU = neonatal intensive care unit. Intended NA included data of women undergoing SA and EA and those who received >1 type of anaesthesia. Intended NA included data of women undergoing SA and EA and those who received >1 type of anaesthesia.

NA was associated with lower odds of NNM, severe neonatal outcome (SNO), Apgar score <7 at 5 minutes after birth, and admission to neonatal intensive care unit (NICU) were observed in both databases. In WHOGS, the odds of END were comparable across the comparison groups (Table 3). In WHOMCS, NA was associated with decreased odds of END in both ante- and intra-partum CS (Table 4). Exposure to NA during CS decreased odds of NNM (pooled OR 0.63; 95% CI 0.55, 0.73), SNO (pooled OR 0.55; 95% CI 0.48, 0.63), Apgar score <7 at 5 minutes after birth (pooled OR 0.35; 95% CI 0.29, 0.43), and NICU admission (pooled OR 0.53; 95% CI 0.45, 0.62) (Fig. 3).

## Discussion

This secondary analysis of two WHO multi-country, facility-based surveys show the odds of severe pregnancy outcomes associated with the various techniques of anaesthesia given for CS. Women undergoing NA for CS had lower risks of MD, MNM, SMO, ICU admission, and PPH than those underwent GA. NA also decreased risks of NNM, SNO, Apgar score <7 at 5 minutes, and NICU admission. As the associations between anaesthesia types and the deaths and near miss of the women and infants are less well examined, these findings are therefore the key information that can provide more evidences on this issue to the existing literature.

Rate of GA for CS varied from 4% in WHOGS to 11.1% in WHOMCS. The pragmatic nature of a cross-sectional study *per se* makes this analysis unable to determine the reason leading to an increased rate of GA for CS in WHOMCS dataset. Our findings of maternal mortalities attributable to GA given during CS of 0.3% in WHOGS and 0.7% in WHOMCS were remarkably higher than that previously reported in a high-income country, where the rate was only 6.5 per million livebirths^9^. A previous systematic review undertaken to determine anaesthesia-attributed deaths of pregnant women in LMICs noted that exposure to GA tripled the odds of maternal death compared with NA (OR 3.3, 95% CI 1.2–9.0)^6^. Difficult airway management, aspiration, and a lack of appropriate monitors are noted to be the leading causes of maternal mortality associated with GA during CS^10^. These findings suggest that in settings with sub-optimal quality care, training and skills, efforts to limit the use of GA for CS may lead to lowering MD in LMICs.

A nationwide, population-based study in Japan reported a reduction of life-threatening complication from a rate of 2.0% of women undergoing GA for CS to 0.7% in women receiving NA^11^. GA performed during CS in LMICs contributes disproportionately to severe maternal morbidity. The rates of MNM were high at 7.7% in WHOGS and 1.0% in WHOMCS. The reduction of the rate of MNM noted in WHOMCS may be secondary to an improvement of healthcare services over time among the facilities in LMICs. Despite this advancement, the harms of GA given during CS in terms of increased risks of MD, MNM, END, and NNM were observed in both ante- and intra-partum CS.

Approximately 4% of women in both WHOGS and WHOMCS were reported to receive more than one anaesthetic techniques during CS. Less clear is whether this was a conversion to GA when insufficient neuraxial technique was considered or intended combination of anaesthesia due to lacking information provided on both datasets. Whilst this group likely represented a small proportion of dataset, previous studies noted a disproportionate high incidence of morbidity among pregnant women receiving multiple anaesthetic techniques during CS^12–14^. To avoid the potential bias, women receiving more than one anaesthetic technique were included and analysed in the group of NA as originally intended NA. The benefits of NA for CS in lowering the risks of MD, MNM, and NNM however remained significant after including women receiving more than one anaesthetic techniques into NA group.

As RCT for assessing the influence of mode of anaesthesia on the mortality and near miss of women and infants is technically impossible because the rarity of these outcomes, the next best option therefore is the large, population-based cohorts^15^. A fundamental limitation of observational study is the potential effect of various confounding factors. Given that the decision to use NA or GA is typically influenced by the type and severity of the indication for CS, these two factors thus are the major confounders when assessing the association between mode of anaesthesia and pregnancy outcomes^15^. This study is no exception. Although a large sample size allowed this study to apply multilevel adjustments to account for clustering effect of CS cases within facility as well as some important potential confounders at individual level; the information regarding the indication of CS however were not available. We attempted to mitigate the effect of the type of indication for CS by applying the timing of CS performed (antepartum and intrapartum CS) as a tentative factor representing the characteristics of CS which may raise the concern of the residual confounding effect. However, benefits of NA in reducing risks of MD and MNM were evident and were highly unlikely to alter the direction of associations when more details of the type and indications of CS were adjusted.

This is a secondary analysis of two large WHO surveys that used pretested, standardized data collection forms collected by well-trained research assistants including a data quality assurance component. Multilevel analysis was applied to account for clustering effect of CS cases within facility. This study applied the outcome definitions and measures according to the standard approach recently recommended by WHO. The large sample size permits this study to determine the associations of anaesthetic techniques and the very infrequent, but devastating occurrences such as MD, MNM, END, and NNM particularly the associations between anaesthesia and END and NNM which have never been reported in the existing literature. Findings of this study can represent the real-life situation and the global perspectives of LMICs as the data were obtained from various LMICs in Africa, Asia, Latin America, and the Middle East.

Some limitations of this study are worthy of consideration in the interpretation of findings. First, this study solely included data obtained from the participating facilities in LMICs. Moreover, both WHOGS and WHOMCS were primarily undertaken in participating facilities with at least 1,000 deliveries per year and were able to provide CS which may harbour an over-representation of complicated pregnancies. This thus limits the generalization to facilities of different backgrounds. Second, this study attempted an adjustment for potential confounders at either individual or facility levels to demonstrate a possible independent association of anaesthetic technique and adverse pregnancy outcomes. However, information on some other variables that might be related to pregnancy outcomes, including adequacy of antenatal care, indications of CS, nutritional status, smoking, type of anaesthesia providers, and obesity were not provided in both datasets and thus residual confounding may remain. However, the effect sizes of the benefits of NA for almost all outcomes were so high that it is quite unlikely to be explained by residual confounding. Moreover, the findings were very much consistent for both WHOGS and WHOMCS. Data collection regarding PPH was different across the two datasets. In WHOGS, only PPH requiring blood transfusion was recorded. In WHOMCS, however, all PPH were recorded but diagnostic decisions for PPH were based on local practices without imposing any definitions of methods and criteria required. This therefore precluded pooling this  data of the two surveys. Finally, while we were able to fully apply the WHO maternal near miss and neonatal near miss criteria in WHOMCS, we used pragmatic definition to identify maternal near miss and neonatal near miss cases in WHOGS because some data for diagnosing these conditions were not completely available in such dataset.

Our analysis of two large multi-country WHO databases in LMICs suggests that the anaesthetic technique used for CS is associated with increased odds of severe maternal and neonatal outcomes in LMICs. NA was associated with decreased odds of deaths and near-miss outcome of either women or infants thus it should be considered as anaesthetic technique of first choice for CS. In addition, limiting the use of GA for CS only when medically necessary may lead to lowering adverse pregnancy outcomes in this setting. As this is a secondary analysis of cross-sectional, observational studies, our findings thus may be hampered by inherent limitations and therefore should be cautiously interpreted.

## Methods

### Setting and design

This is a secondary analysis of two WHO multi-country, facility-based surveys. The WHO Global Survey on Maternal and Perinatal Health (WHOGS) included 373 health facilities in 24 countries, in Africa and Latin America (2004–2005), and Asia (2007–2008). The WHO Multi-country Survey on Maternal and Newborn Health (WHOMCS) conducted during 2010–2011, included 359 health facilities in 29 countries in Africa, Asia, Latin America, and the Middle East. Details of the methodologies of these surveys were published elsewhere^7,8^. In brief, for the WHOGS, countries and health facilities were randomly selected by using stratified multistage cluster sampling approach. In each country, the capital city and two randomly selected provinces (probability proportional to population) were sampled. Seven facilities with capacity to perform CS and over 1000 deliveries per year were randomly selected from each province. The WHOMCS built on the existing WHOGS network of health facilities. WHOGS countries were invited to participate in the WHOMCS; two countries (Cuba and Algeria) were unable to participate. Within the remaining 22 countries, 32 facilities with very poor recruitment, data quality issues, or being unable to participate were not included in the WHOMCS. To improve global representation, seven new countries were added with a total of 29 countries in Africa, Asia, Latin America, and the Middle East included.

The study population of the WHOGS and WHOMCS were women who delivered their babies at the participating facilities during the study period. Data of individual women and their deliveries from time of presentation at the facility until discharge, death or the seventh day post-partum (whichever occurred first) were extracted from the facility medical records and recorded by trained data collectors into individual forms especially created for the surveys. Data were completed after delivery and before hospital discharge of each woman. There was no direct contact between data collections and women. Outcomes occurring after discharge or during subsequent re-admissions were not captured.

### Study population

We included singleton pregnancies delivered by CS in 21 LMICs common to both surveys. The entire list of countries included in the analysis can be found as Supplementary Table S1. Technique of anaesthesia given for CS were categorized as GA, NA which included SA and EA, and more than one technique. A CS that was recorded to use more than one anaesthetic technique was classified as receiving more than one technique. For “more than one technique”, data was not available to indicate whether this was a conversion of NA to GA, or a planned combination of both types of anaesthesia. With the assumption that these patients most likely received a NA before conversion to GA, and not the reverse, we then combined data of women who received more than one anaesthetic technique to those with NA to represent an originally intended NA.

We excluded women who delivered by CS without information on the anaesthetic technique. In the analyses of neonatal outcomes, we excluded cases with abortion (birthweight < 500 g or gestational age <22 weeks) and those with congenital malformations. We also excluded cases with macerated stillbirths as this outcome was very unlikely to be the effect of anaesthesia given for caesarean section.

### Outcome measures and definitions

Adverse pregnancy outcomes were categorized into maternal and neonatal outcomes. Adverse maternal outcomes included MD (death of mother during admission, up to 7 days postpartum or discharge, whichever occurred first), maternal near miss (MNM, a woman who nearly died but survived a complication that occurred during pregnancy, childbirth or up to 7 days postpartum), severe maternal outcome (SMO) which is a combination of MD and MNM, admission to intensive care unit (ICU) and postpartum haemorrhage (PPH). Maternal near miss cases were identified according to the WHO maternal near miss criteria in the WHOMCS study^8^. In the WHOGS survey, we used a pragmatic definition in which a woman was classified as near miss if she experienced one or more of the following: hysterectomy, blood transfusion, admission to ICU and eclampsia^16^. In WHOGS, only PPH requiring blood component transfusion was recorded. In WHOCS, the diagnosis of PPH was based on local practices without imposing any definition and criteria required. Adverse neonatal outcomes included early neonatal death (END) which was defined as death of liveborn up to the 7^th^ day postpartum or discharge, whichever occurred first, neonatal near miss (NNM), severe neonatal outcome (SNO), Apgar score <7 at five minutes and admission to neonatal intensive care unit (NICU). Definition of near miss and severe outcome used for maternal outcome were applied for neonatal near miss and severe neonatal outcome^17,18^.

The selection of factors to be adjusted in this study was based on the literature review. Potential confounders were considered at both the facility and individual levels. Potential confounders at the individual level included maternal age, educational attainment, marital status, parity, gestational age, type of CS (antepartum or intrapartum CS) and comorbidities including preeclampsia or eclampsia, underlying diseases (heart disease, lung disease, renal disease, malaria, severe anaemia, and chronic hypertension), and type of anaesthesia provider (available only in WHOGS dataset). At the facility level, it was the facility capacity index (FCI). The development and application of FCI has been described elsewhere^19,20^. In this analysis, anaesthesia resource was excluded from the FCI, so its effects could be determined separately. The list of abbreviations can be found as Supplementary Table S2.

### Statistical analysis

The prevalence of each anaesthetic technique was calculated for each survey. Characteristics of women and newborns were described in frequency and percentage. For each database, two-level logistic regression analysis was performed to adjust clustering effects of health facilities and investigate risks of adverse maternal and neonatal outcomes in women undergoing CS by different anaesthetic using lme4 package in R software^21,22^. The risk for adverse maternal and neonatal outcomes associated with type of anaesthesia were presented by adjusted OR with corresponding 95% CIs.

We applied the two‐stage statistical approach for individual participant data (IPD) meta‐analysis^23^, because this approach is allowed to adjust all potential confounders that were available for each dataset. We started by analysing the IPD separately from WHOGS and WHOMCS to obtain the aggregate adjusted ORs for the adverse outcomes. We, then, combined the adjusted ORs of the two datasets using a random effects model described by Der Simonian and Laird^24^.

### Ethics approval and consent to participate

The study protocols of WHO Global Survey on Maternal and Perinatal Health and WHO Multi- country Survey on Maternal and Newborn Health Committee and the relevant ethical clearance mechanisms in all countries were approved by the WHO Ethical Review Committee. This study adhered to the principles of the Declaration of Helsinki. Informed consent was formally waived by the WHO Ethical Review committee. Therefore, written consent from individual women was not required as data collectors extracted data from medical records and did not contact the individual women.

## Supplementary information

Supplementary Information.

## Data Availability

The datasets generated and/or analysed during the current study are not publicly available because they belonged to Department of Sexual and Reproductive Health and Research, The World Health Organization but could be available from WHO on reasonable request.
