# Supplementary material for: Outcomes associated with anaesthetic techniques for caesarean section in low- and middle-income countries: a secondary analysis of WHO surveys
Source: Sci Rep. 2020 Jun 23;10:10176. doi: 10.1038/s41598-020-66897-8 (PMC7311389; doi:10.1038/s41598-020-66897-8)
Supplement: Supplementary file 1 — Supplementary Information. [file 41598_2020_66897_MOESM1_ESM.pdf]

Supplementary information for

Outcomes associated with anaesthetic techniques for caesarean section in low- and middle-income countries: a secondary analysis of WHO surveys

Pisake Lumbiganon[1], Hla Moe[2], Siriporn Kamsa-ard[3], Siwanon Rattanakanokchai[3], Malinee Laopaiboon[3], Chumnan Kietpeerakool[1,*], Nampet Jampathong[4] Monsicha Somjit[5], José Guilherme Cecatti[6], Joshua P. Vogel[7], Ana Pilar Betran[7], Suneeta Mittal[8], Maria Regina Torloni[9]

[*]Correspondence: Chumnan Kietpeerakool, Department of Obstetrics and Gynaecology, Faculty of Medicine, Khon Kaen University, 123 Mitraparb Road, Amphur Muang, Khon Kaen, Thailand, 40002 (Tel: +66815935700, Email: kiet_ji@hotmail.com)

Supplementary table S1. List of countries included in the analysis

1. Angola
2. Argentina
3. Brazil
4. Cambodia
5. China
6. Democratic Republic of the Congo
7. Ecuador
8. India
9. Kenya
10. Mexico
11. Nepal
12. Nicaragua
13. Niger
14. Nigeria
15. Paraguay
16. Peru
17. Philippines
18. Sri Lanka
19. Thailand
20. Uganda
21. Viet Nam

Supplementary table S2. List of abbreviations

CS	Caesarean Section
EA	Epidural Anaesthesia
END	Early Neonatal Death
FCI	Facility Capacity Index
GA	General Anaesthesia
ICU	Intensive Care Unit
IPD	Individual Participant Data
LMICs	Low- And Middle-Income Countries
MD	Maternal Deaths
MNM	Maternal Near Miss
NA	Neuraxial Anesthesia
NICU	Neonatal Intensive Care Unit
NNM	Neonatal Near Miss
PPH	Postpartum Haemorrhage
RCTs	Randomised Controlled Trials
SA	Spinal Anaesthesia
SMO	Severe Maternal Outcome
SNO	Severe Neonatal Outcome
WHO	World Health Organization
WHOGS	World Health Organization Global Survey
WHOMCS	World Health Organization Multi-Country Survey
